# Folded Synthetic Peptides and Other Molecules Targeting Outer Membrane Protein Complexes in Gram-Negative Bacteria

**DOI:** 10.3389/fchem.2019.00045

**Published:** 2019-02-06

**Authors:** John A. Robinson

**Affiliations:** Department of Chemistry, University of Zurich, Zurich, Switzerland

**Keywords:** antibiotic, gram-negative bacteria, lipopolysaccharides (LPS), Lpt complex, Bam complex, ß-hairpin mimetics, ß-barrel, ß-jellyroll

## Abstract

Conformationally constrained peptidomimetics have been developed to mimic interfacial epitopes and target a wide selection of protein-protein interactions. ß-Hairpin mimetics based on constrained macrocyclic peptides have provided access to excellent structural mimics of ß-hairpin epitopes and found applications as interaction inhibitors in many areas of biology and medicinal chemistry. Recently, ß-hairpin peptidomimetics and naturally occurring ß-hairpin-shaped peptides have also been discovered with potent antimicrobial activity and novel mechanisms of action, targeting essential outer membrane protein (OMP) complexes in Gram-negative bacteria. This includes the Lpt complex, required for transporting LPS to the cell surface during OM biogenesis and the BAM complex that folds OMPs and inserts them into the OM bilayer. The Lpt complex is a macromolecular superstructure comprising seven different proteins (LptA-LptG) that spans the entire bacterial cell envelope, whereas the BAM complex is a folding machine comprising a ß-barrel OMP (BamA) and four different lipoproteins (BamB-BamE). Folded synthetic and natural ß-hairpin-shaped peptides appear well-suited for interacting with proteins within the Lpt and BAM complexes that are rich in ß-structure. Recent progress in identifying antibiotics targeting these complexes are reviewed here. Already a clinical candidate has been developed (murepavadin) that targets LptD, with potent antimicrobial activity specifically against pseudmonads. The ability of folded synthetic ß-hairpin epitope mimetics to interact with ß-barrel and ß-jellyroll domains in the Lpt and Bam complexes represent new avenues for antibiotic discovery, which may lead to the development of much needed new antimicrobials to combat the rise of drug-resistant pathogenic Gram-negative bacteria.

## Introduction

Mimics of protein epitopes mediating macromolecular interactions have attracted great interest in chemical biology and drug design. One of the early drivers of protein epitope mimetic design was in targeting protein-protein interactions (PPIs) (Arkin et al., 2014). It has become clear, however, that epitope mimetics may have many other uses, such as targeting protein-nucleic acid interactions, the active sites of enzymes, and membrane bound receptors, to name but a few (Robinson, 2008; Zerbe et al., 2017). The structure-based design of protein epitope mimetics is often focused on the secondary and tertiary structures involved in biomolecular interactions, including α-helical, ß-turn, ß-strand, and ß-hairpin conformations (Bullock et al., 2011; Watkins and Arora, 2014). A diverse variety of molecular scaffolds for mimetic design has been explored, including conformationally constrained peptides that maintain some or all of the backbone and amino acid side-chain groups of the epitope. Naturally occurring, highly stable mini-protein scaffolds may also be engineered to display target epitope(s) (Wuo and Arora, 2018). The approach is not limited to peptides, however, as illustrated by rigid or constrained organic scaffolds that can display side chain groups important for protein recognition (Gopalakrishnan et al., 2016). With synthetic epitope mimetics, the possibility exists to harness the robust tools of synthetic chemistry to optimize properties, such as target affinity and selectivity, as well as pharmacological and related drug-like (ADMET) properties (Morrison, 2018).

In this focused review, the application of ß-hairpin-shaped peptides and peptidomimetics in the field of antibiotic research is highlighted, in particular, molecules with new antimicrobial mechanisms of action targeting outer membrane (OM) biogenesis in Gram-negative bacteria. This research is set against the backdrop of the growing global health threat caused by the rapid evolution and spread of antibiotic resistance, particularly in Gram-negative bacteria such as *Klebsiella pneumoniae, Acinetobacter baumanii, Pseudomonas aeruginosa*, and *Enterobacter sp*. (Rice, 2008; Boucher et al., 2009; WHO, 2017) as well as the great difficulties encountered in discovering new antibiotics with novel mechanisms of action.

## Outer Membrane Biogenesis

**Figure 1:**
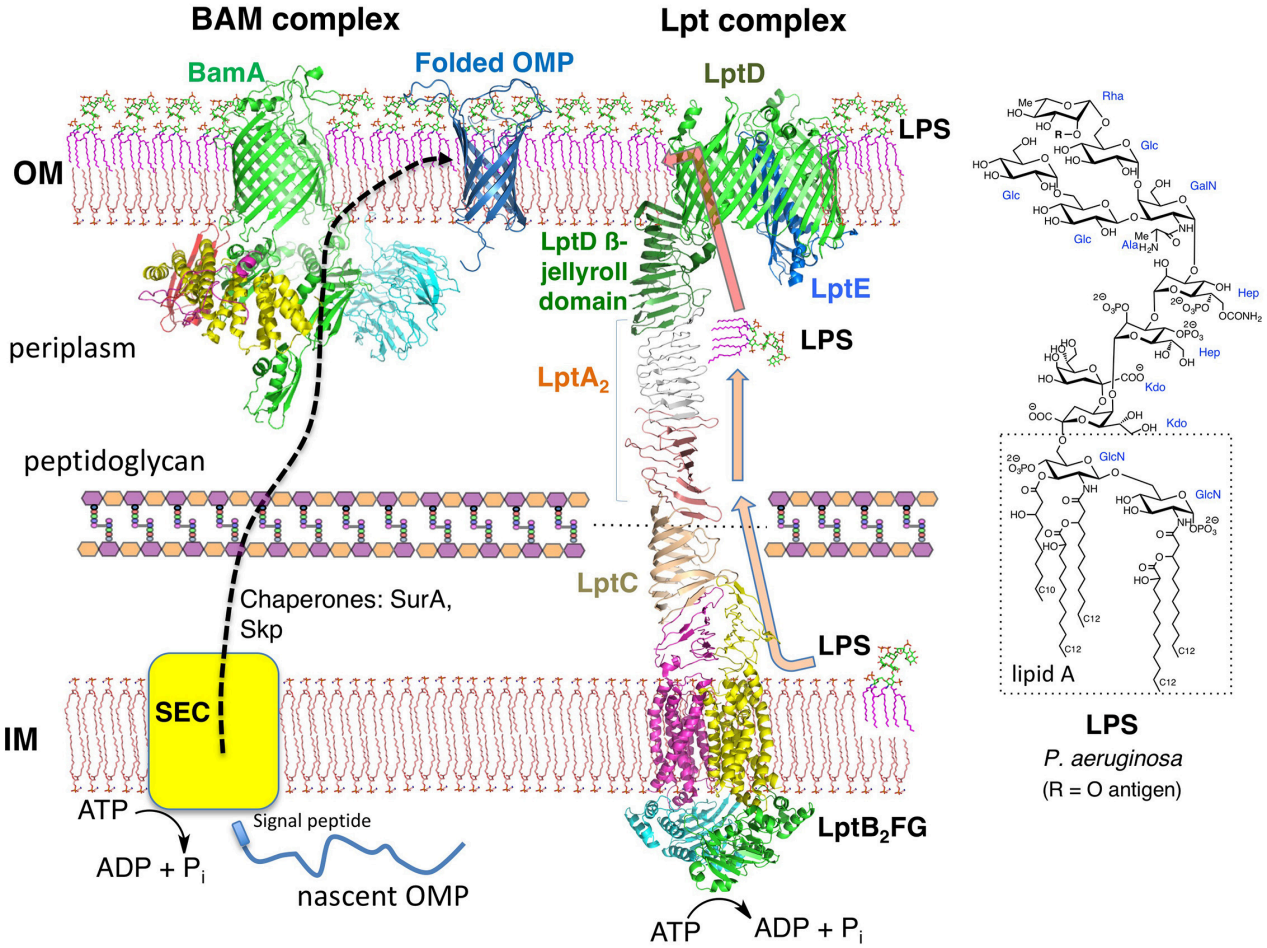
The Gram-negative envelope. The OM is an asymmetric bilayer, while the IM is a symmetric bilayer composed mainly of glycerophospholipids. The BAM complex (BamABCDE) functions as a ß-barrel folding catalyst, whereas the Lpt complex (LptAB_2_CDEFG, LptA dimer shown) is essential for LPS transport to the OM. Crystal structures from the PDB of the BAM complex and individual components of the Lpt complex were used to make the figure. The structure of a typical LPS from *P. aeruginosa* is shown.

Gram-negative bacteria possess two membranes around the cytoplasm. The inner membrane (IM) is a typical symmetric glycerophospholipid bilayer, whereas the OM is an asymmetric bilayer containing an inner leaflet of glycerophospholipid and an outer leaflet containing lipopolysaccharide (LPS) (Henderson et al., 2016; May and Silhavy, 2017). The periplasm is an aqueous compartment between the IM and OM, containing the peptidoglycan cell wall (Figure 1). Both the IM and the OM harbor many anchored lipoproteins (Narita and Tokuda, 2017), as well as numerous integral membrane proteins. In the IM, these integral membrane proteins contain multiple transmembrane helical domains, whereas those in the OM, with one known exception, fold into transmembrane ß-barrel domains (Ranava et al., 2018). LPS is a complex glycolipid with many known structural variants, but with a common lipidated and phosphorylated bis-glucosamine core called lipid-A (Figure 1; Raetz et al., 2007). The phosphate groups in lipid A and inner core regions of neighboring LPS molecules coordinate to Mg^2+^ or Ca^2+^ ions, thereby strengthening the OM, which is important for OM function and stability. It was shown recently that not only the peptidoglycan layer but also the OM contributes as a load-bearing structure with mechanical strength to help resist osmotic forces occurring across the cell envelope (Rojas et al., 2018). The outer oligosaccharide segment of LPS may contain over a hundred sugar residues, including the so-called O-antigen responsible for characteristic immunological serotypes. The combined IM and OM represent a formidable permeability barrier, with hydrophobic molecules unable to penetrate the OM unless they can gain access through OM porins, and most hydrophilic molecules unable to cross the IM, again unless specific transporters are available (Nikaido, 2003). For those antibiotics that are able to gain entry to the cytoplasm, a formidable array of drug export pumps has evolved in Gram-negative bacteria to pump antibiotics across both membranes and back out of the cell (Poole, 2005; Masi et al., 2017).

The unusual architecture of the OM does not arise spontaneously. Important progress has been made recently in understanding how LPS is transported from its site of biosynthesis at the IM to the cell surface during growth (Konovalova et al., 2017). LPS transport to the cell surface is mediated by seven lipopolysaccharide transport (Lpt) proteins (LptA-LptG) that assemble into a macromolecular complex spanning the cell envelope (Figure 1) (Freinkman et al., 2012; May et al., 2015; Simpson et al., 2015; Okuda et al., 2016; Sherman et al., 2018). The entire protein complex must form before LPS transport can begin. The 3D structures of all seven Lpt proteins, from various Gram-negative bacteria, have now been solved (Suits et al., 2008; Tran et al., 2010; Dong et al., 2014, 2017; Qiao et al., 2014; Bollati et al., 2015; Botos et al., 2016). A computer model representing the entire Lpt complex is shown in Figure 1. The IM adenosine 5'-triphosphate (ATP)-binding cassette transporter LptFGB_2_ associates with the membrane anchored LptC and uses ATP hydrolysis in the cytoplasm to power the extraction of LPS from the outer leaflet of the IM and transfer to LptC. Subsequently, LPS molecules are pushed over the periplasm across a bridge formed by LptA (Okuda et al., 2012; Luo et al., 2017). The LptA bridge, possibly as a monomer or as an oligomer (LptA_n_), interacts with LptC in the IM and with the LptD/E complex anchored in the OM (Freinkman et al., 2012). The essential function of the LptD/E complex is to receive LPS molecules coming across the LptA bridge and translocate them into the outer leaflet of the OM. Much experimental evidence has now accrued in support of the so-called PEZ-model (in analogy to the candy dispenser) of LPS transport, in which ATP hydrolysis within the LptB_2_ dimer powers LPS extraction from the IM (Okuda et al., 2016; Sherman et al., 2018). With each power stroke, LPS molecules are pushed across the LptA bridge toward LptD/E in the OM, and eventually onto the cell surface. During exponential growth, the flux of LPS through the Lpt pathway is estimated to be ≈1,200 molecules s^−1^ (Lima et al., 2013).

Almost all bacterial outer membrane proteins (OMPs) fold into transmembrane ß-barrel domains, with their N and C termini facing the periplasm. The C-terminal region of LptD contains one of the largest ß-barrels so far characterized, with 26 ß-strands integrated into the OM bilayer (Figure 1; Dong et al., 2014; Qiao et al., 2014; Botos et al., 2016). Importantly, the N-terminal segment of LptD is located in the periplasm and contains a ß-jellyroll domain. The same highly conserved ß-jellyroll fold is also present in the soluble periplasmic protein LptA, and in membrane-anchored LptC (Suits et al., 2008; Tran et al., 2010; Laguri et al., 2017). The V-shaped sides of the ß-jellyroll comprise 16 antiparallel ß-strands that possess a twisted hydrophobic internal channel suitable for interacting with the fatty acyl chains of LPS, whilst leaving the polar sugar residues of LPS exposed to solvent (Villa et al., 2013). The ß-jellyrolls in LptC-LptA-LptD associate through PPIs. *In-vitro* binding studies have shown that individual LptA-LptA and LptA-LptC ß-jellyrolls interact with binding constants in the low to sub-micromolar range (Merten et al., 2012; Schultz et al., 2017). Alignment of the V-shaped grooves formed by association of the ß-jellyrolls in LptC, LptA, and LptD, therefore, provide a path for LPS to be shuttled across the periplasm to the LptD/E complex in the OM (Okuda et al., 2016; Laguri et al., 2017).

Given its essential role for OM biogenesis in Gram-negative bacteria and its in-part exposed position on the cell surface, it is perhaps remarkable that during the golden age of antibiotic discovery in the latter half of the twentieth century, no antibiotics were reported that target the Lpt complex.

## Molecules Targeting the Lpt Complex

**Figure 2:**
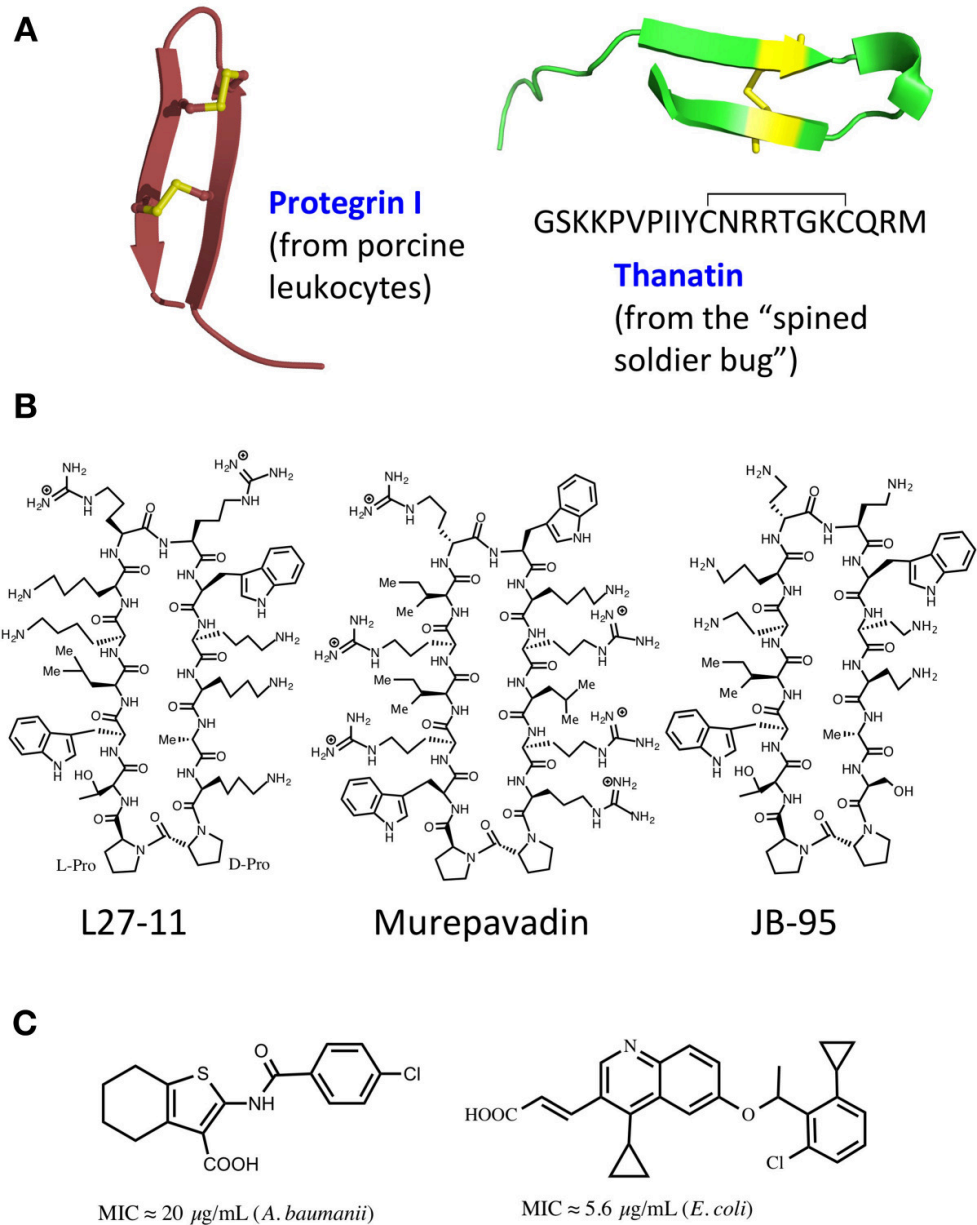
Natural and synthetic antibiotics**. (A)**, Ribbon representations of protegrin I (PDB 1PG1) and thanatin (8TFV). Disulfide bonds shown in yellow. **(B)**, Macrocyclic peptidomimetic antibiotics. **(C)**, Molecules targeting MsbA.

The ß-hairpin secondary structure is found in many naturally occurring cationic antimicrobial peptides (CAMPs) produced by the innate immune systems in a wide variety of organisms (Panteleev et al., 2017). Some disulfide cross-linked CAMPs have amphipathic hairpin structures, which result in an ability to disrupt biological membranes by pore formation (class-I). For example, protegrin I (PG-I) (Figure 2) isolated from porcine leukocytes, shows potent broad-spectrum antimicrobial activity in the micromolar range, and disrupts membranes in both Gram-positive and Gram-negative bacteria, as well as in eukaryotes (Zhao et al., 1994). Both enantiomers of PG-I are equally active as antibiotics, due to the target being the lipid bilayer of the cell membrane (Wang et al., 2016). However, other naturally occurring hairpin CAMPs do not disrupt bacterial membranes and their antimicrobial activity shows a high enantioselectivity (class-II), which points to a quite different mechanism of action (MoA).

Interestingly, the first reported example of a hairpin-shaped cationic antimicrobial peptide targeting the Lpt complex arose from efforts to produce structural mimics of the class-I CAMP protegrin I (Shankaramma et al., 2002; Robinson et al., 2005). The macrocyclic peptide called L27-11 (Figure 2) was derived by iterative optimization of ß-hairpin mimetics, that led from an initial hit with broad-spectrum antimicrobial activity and micromolar minimal inhibitory concentrations (MICs), to nanomolar activity against Gram-negative pseudomonads, in particular *P. aeruginosa* (Srinivas et al., 2010). L27-11 does not lyse bacterial membranes and the enantiomer is inactive. Structure-activity studies showed that the folded ß-hairpin conformation of the peptide is critical for its antimicrobial activity (Schmidt et al., 2013; Vetterli et al., 2016). Further, optimization of drug-like properties led to a clinical candidate called murepavadin (also called POL7080) (Figure 2). Murepavadin is currently undergoing phase III clinical studies to treat lung infections caused by *Pseudomonas aeruginosa* (Martin-Loeches et al., 2018; Wach et al., 2018), which is recognized as a critical priority 1 pathogen by the WHO (2017).

The identification of the target for L27-11 and murepavadin in *Pseudomonas* spp. as LptD came from photoaffinity labeling studies and the analysis of spontaneously drug-resistant *P. aeruginosa* strains, which could be isolated at low frequency (Srinivas et al., 2010). The resistant isolates typically contained mutations in the periplasmic ß-jellyroll domain of LptD, and the mutant allele was able to confer resistance to the antibiotic when introduced into the sensitive wild-type strain. Further mechanistic studies provided evidence that L27-11 indeed causes inhibition of LPS transport to the cell surface in *P. aeruginosa*, as implied from its interaction with LptD (Werneburg et al., 2012).

The unique specificity of these antibiotics for *Pseudomonas* spp. arises from structural differences between LptD within Gram-negative bacteria. The crystal structure of a truncated LptD from *P. aeruginosa* revealed the C-terminal ca. 600 residue ß-barrel domain, which is highly conserved in LptD from all Gram-negative bacteria (Botos et al., 2016). So far, no 3D structure is available for the periplasmic segment of LptD from pseudomonads. However, the ß-jellyroll domain is present and highly conserved, consistent with its key interaction with LptA and function as a landing stage for LPS molecules crossing the periplasmic bridge. However, the periplasmic domain of LptD in pseudomonads is longer than in most other γ-proteobacteria, due to an extra domain of ca 100 residues at the N-terminus. The structure and function of this insert domain is currently unknown. Photolabeling studies showed recently that the antibiotics L27-11 and murepavadin interact at a site in LptD close to both the ß-jellyroll and the insert domain (Andolina et al., 2018). Moreover, mutations in LptD that give rise to spontaneous resistance to the antibiotics (Srinivas et al., 2010), discussed above, lie within the ß-jellyroll domain. These observations narrow down the binding site for the antibiotic in LptD to the periplasmic domains, provide an explanation for the unique selectivity of the antibiotic, and for its ability to interfere with LPS transport to the cell surface.

There is now considerable interest in discovering new inhibitors of the LPS transport pathway, as potential antibiotics against Gram-negative bacteria. Many inhibitors of the LPS biosynthetic enzyme LpxC, which catalyzes the first committed step in LPS biosynthesis, have been reported over the past 20 years (Erwin, 2016). However, LpxC is a soluble enzyme for which a convenient activity assay is available. In the case of LPS transport, a convenient assay for inhibitor discovery has been less straightforward to develop, given that the seven-protein trans-envelope Lpt complex connects two membranes and contains multiple integral membrane proteins as well as the soluble periplasmic protein LptA. However, an ingenious solution to this problem was reported recently, by reconstituting membrane-to-membrane transport of LPS *in-vitro* using two different proteoliposomes, one containing the purified LptB_2_FGC complex (IM proteoliposomes) and the other containing the LptD/E complex (Sherman et al., 2018). Addition of soluble LptA to create a bridge between the IM and OM proteoliposomes enabled LPS transport, driven by ATP hydrolysis, as monitored by cross-linking of LPS to photoactive sites in Lpt components. More recent mechanistic studies have revealed in this fully reconstituted *in-vitro* system a mechanism for coupling between the OM LptD/E translocon and ATP hydrolysis at the IM (Xie et al., 2018). This coupling appears to allow the LptD/E translocon to call a halt to ATP hydrolysis and LPS transport once a critical concentration of LPS is achieved in the OM.

A different cell-based screen for LPS biosynthesis and transport has also been described recently and used to identify inhibitors of an earlier step of LPS transport, namely that mediated by MsbA, an integral IM protein (Zhang et al., 2018). MsbA is a member of the ABC ATP-dependent transporter superfamily that mediates an earlier step of LPS transport, by flipping core LPS from its site of synthesis on the inner leaflet to the periplasmic side of the IM (Ward et al., 2007; Mi et al., 2017). The screen made use of engineered *Acinetobacter baumanii* strains lacking efflux pumps and LPS-modifying enzymes and comparing effects on growth compared to the WT strain. The best identified inhibitors showed MICs in the low micromolar range and contained a tetrahydrobenzothiophene scaffold (Figure 2C). Mechanistic studies revealed that these molecules bind to MsbA, stimulating the ATPase activity while decoupling it from LPS translocation. This leads to elevated levels of LPS at the IM, which has a deleterious effect on the membrane. In a different study, a high throughput *in-vitro* assay with purified MsbA led to the identification of a family of quinoline derivatives that specifically inhibit the activity of MsbA on cells (Figure 2C; Alexander et al., 2018; Ho et al., 2018). These molecules typically inhibit the growth of *E. coli* and *Klebsiella pneumoniae* with MICs in the low micromolar range. Crystal structures were also obtained of *E. coli* MsbA with bound quinoline inhibitors, which show the protein trapped in an inward-facing LPS-bound conformation. Unfortunately, the hydrophobic nature of these active quinolines correlated with high levels of plasma protein binding and a significant loss of antimicrobial activity in the presence of serum, indicating non-optimal drug-like properties (Alexander et al., 2018). Nevertheless, these studies validate MsbA as an antibacterial target and establish screening methods that can be used to discover new LPS transport inhibitors.

A stimulator of the ATPase LptB, the IM component of the Lpt complex that powers the seven protein Lpt complex and LPS transport (Figure 1), has also been identified recently (May et al., 2017; Mandler et al., 2018). Novobiocin belongs to a family of antibiotics largely directed against aerobic Gram-positive organisms, which function by blocking the ATPase activity of the B subunit of DNA gyrase. Novobiocin has lower activity against Gram-negative bacteria due to the permeability barrier posed by the OM. However, using an *E. coli* strain with a leaky OM, it was shown that novobiocin also binds and stimulates LptB, leading to improved LPS transport (May et al., 2017). A crystal structure revealed that novobiocin binds at a critical position at the LptB-LptFG interface. This binding may facilitate one or more steps in the catalytic cycle of the ATPase, coordinating nucleotide binding with LPS release. Novobiocin contains a substituted coumarin nucleus. An analog that retains LptB-stimulatory activity but is unable to inhibit DNA gyrase, although not toxic on its own, acts synergistically with polymyxin against Gram-negative bacteria (Mandler et al., 2018). With the new insights gained from these studies, it may become possible to discover new derivatives of novobiocin that inhibit rather than activate the LPS transporter.

The synthetic peptidomimetic antibiotics described earlier were until recently the only compounds known to target the Lpt complex and inhibit LPS transport in pseudomonads. Their discovery not only validated the Lpt complex as a valuable target in antibiotic discovery but raised the intriguing question whether antimicrobial natural products might exist with a similar mechanism of action. The first example of a natural product targeting the Lpt complex in *E. coli* was reported recently, namely the cationic antimicrobial peptide thanatin (Figure 2; Vetterli et al., 2018). Thanatin was first isolated from the hemipteran insect *Podisus maculiventris* (spined soldier bug) (Fehlbaum et al., 1996), and shows antimicrobial activity against several Gram-negative bacteria with MICs < 1.5 μM. Although no activity is seen against *Staphylococcus aureus*, thanatin is also active against some other Gram-positive bacteria with MICs ≈1–5 μM. Of special interest is the observation that the enantiomeric form (D-thanatin) loses much of its activity against all the Gram-negative strains tested, indicating a likely chiral target.

Evidence that thanatin interacts with the Lpt complex came again from *in-vivo* photoaffinity interaction mapping with *E. coli*. Potential interaction partners were identified following photolabeling, using a powerful mass spectrometry (MS)-based proteomic analysis. Three photolabeled OMPs revealed by this study were LptD, LptA, and BamB, of which LptD and LptA were the most significantly labeled (Vetterli et al., 2018). A second line of evidence implicating LptA as a target for thanatin came from analysis of spontaneous thanatin-resistant *E. coli* (Than^R^) mutants. Of several Than^R^ mutants isolated, all contained mutations in LptA, and the mutated *lptA* gene when introduced into wild-type *E. coli* could confer resistance to the antibiotic. Indirect evidence that thanatin inhibits LPS transport was provided by transmission electron microscopy (EM) of thanatin-treated E. coli cells, which revealed a major perturbation to the membrane architecture, and the accumulation of membrane-like material inside the cell, which is typical of effects seen when individual components of the Lpt complex are down-regulated. However, direct evidence for an effect on LPS transport might be forthcoming with the emergence of the powerful *in-vitro* assays discussed above.

**Figure 3:**
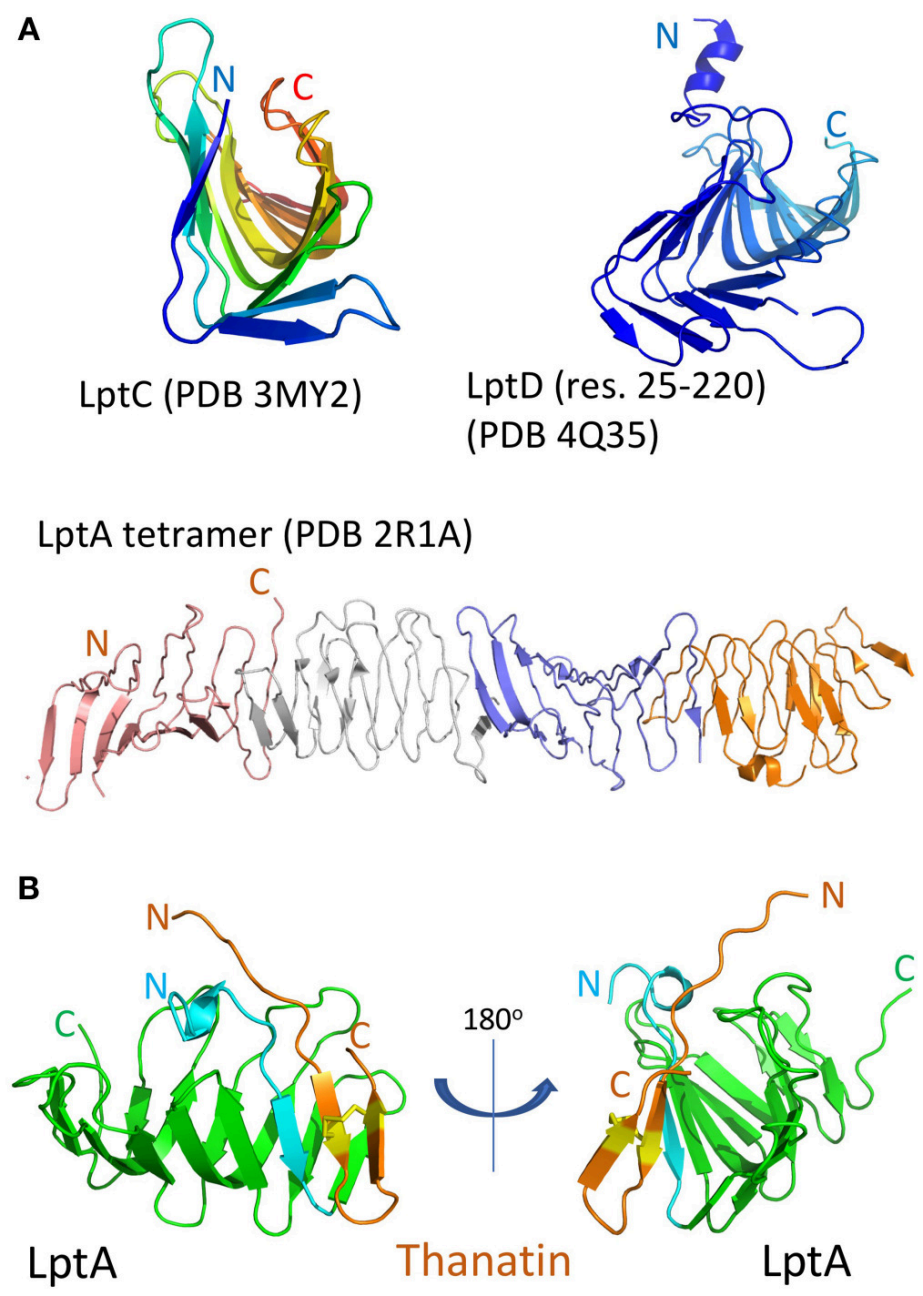
The ß-jellyroll fold. **(A)**, Crystal structures of an LptA oligomer, LptC, and the N-terminal jellyroll domain in LptD (res. 25–220). **(B)**, NMR structure of the thanatin-LptA complex in two orientations (PDB 6GD5). The thanatin hairpin (orange, with disulfide bond in yellow) interacts with the N-terminal ß-strand (light blue) of LptA, at a site used for interactions with other LptA molecules and with LptC, while a structurally conserved site is also present in the N-terminal jellyroll domain of LptD (see Figure 1).

LptD is a large ß-barrel membrane protein, whereas LptA is a small soluble periplasmic protein, so why should thanatin interact with both? Despite this major difference in size and location, both LptD and LptA, as well as LptC, all contain structurally homologous ß-jellyroll domains, which mediate formation of the Lpt trans-periplasmic bridge (Figure 3). The crystal structure of an *E. coli* LptA oligomer revealed how LptA subunits interact in a head-to-tail fashion through their N-and C-terminal ß-strands, by ß-strand complementation (Suits et al., 2008). In analogy, the C-terminal strands of the jellyroll in LptC should interact with the N-terminal strands in LptA, and the C-terminal region of LptA interacts with the N-terminal ß-strands in the jellyroll in LptD (Tran et al., 2010).

*In-vitro* assays revealed that thanatin binds to both recombinant LptA and the LptD/E complex, each in the low nanomolar range. An NMR solution structure of the thanatin-LptA complex showed how the N-terminal ß-strand of the thanatin hairpin docks in a parallel orientation onto the first N-terminal ß-strand in the LptA jellyroll (Figure 3B; Vetterli et al., 2018). The second ß-strand of thanatin is mostly solvent exposed, although key side chains make contact with hydrophobic pockets on LptA. An array of hydrophobic, pi-pi-stacking, polar hydrogen-bonding and charge-charge electrostatic interactions appear to be involved in stabilizing the complex. Moreover, the thanatin binding site in the N-terminal strand of LptA overlaps the site used for LptA-oligomer formation, for the interaction of LptA with LptC, and for the interaction of LptA with LptD. Modeling studies suggest that the thanatin binding site in LptA should be highly conserved in the jellyroll at the N-terminus of LptD (Figure 3). These results suggest that thanatin should inhibit multiple PPIs, including those between LptC-LptA, LptA-LptA, and LptA-LptD, required for assembly of the trans-periplasmic Lpt bridge. To conclude, these results highlight a new paradigm for an antibiotic action, targeting a network of PPIs required for the assembly of the Lpt complex in *E. coli*.

The LptA-thanatin complex reveals how the ß-hairpin fold in thanatin is well-suited for binding to a protein through an exposed ß-strand, a mechanism called ß-augmentation (Remaut and Waksman, 2006). Interestingly, ß-strand augmentation is believed to occur in other complexes that bind unfolded OMPs. Of special interest are components of the ß-barrel folding machine (BAM), which catalyzes the folding and insertion of unfolded ß-barrel proteins into the OM during OM biogenesis. Exposed ß-sheet edges in both BamA and BamB have been implicated in peptide binding by ß-strand augmentation (Kim et al., 2007; Heuck et al., 2011). As mentioned above, it is interesting to note that a third OMP identified by photoaffinity interaction mapping with thanatin was BamB. However, so far it is not known whether thanatin can bind *in-vitro* to BamB or other members of the BAM complex.

## Molecules Targeting the BAM Complex

The ß-barrel OMP LptD is essential for the biogenesis of the OM. ß-Barrel OMPs, however, are also dependent on specialized machinery in the OM to catalyze their folding and insertion into the OM. After biosynthesis in the cytoplasm, newly expressed unfolded OMPs are exported across the IM using the Sec translocon and escorted across the periplasm bound to dedicated chaperones, before delivery to the BAM complex in the OM (Figure 1; Hagan et al., 2011). The BAM complex, comprising the ß-barrel OMP BamA and four lipoproteins BamB-BamE, catalyzes the folding and insertion of ß-barrel OMPs into the OM. Indeed, LptD is an important substrate of the BAM complex. It has been estimated that folding, OM insertion and disulfide oxidation of an LptD molecule into the OM requires about 20 min, a remarkably long time given that *E. coli* doubles during exponential growth every ≈20–40 min (Lee et al., 2016).

**Figure 4:**
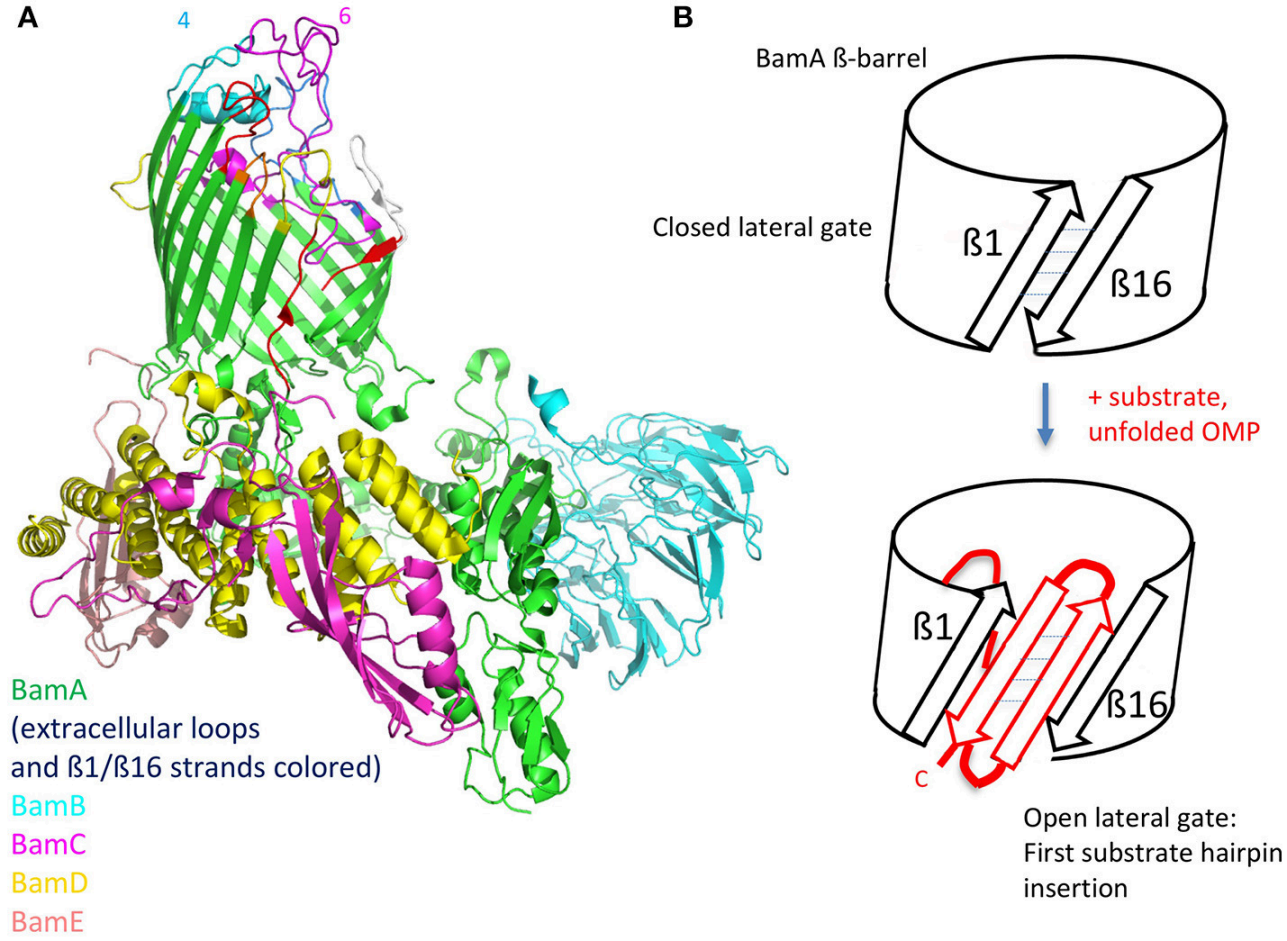
The BAM complex**. (A)**, Structure of the entire BamABCDE complex determined by cryo EM (PDB 5LJO). The ß1 and ß16 strands in the BamA ß-barrel are colored red in an open conformation. The external loops on BamA are also colored, with loop-4 and loop-6 indicated. **(B)**, Depiction of a possible folding mechanism in which the ß1-ß16 lateral gate in the BamA ß-barrel opens and acts as a template for binding the first C-terminal ß-strand of the precursor OMP. Repeated further opening and hairpin insertions lead to folding and finally budding out of the complete ß-barrel (see text for further details).

The structures of individual BAM subunits, sub-complexes as well as the entire BAM complex have been solved by both X-ray crystallography and by cryo-EM (Bakelar et al., 2016; Gu et al., 2016; Han et al., 2016; Iadanza et al., 2016). BamA contains a 16-stranded ß-barrel and five N-terminal periplasmic polypeptide transport-associated (POTRA) domains that interact with the four lipoproteins (BamB-E) forming a ring-like structure underneath the ß-barrel (Figure 4). BamA and BamD are the only essential components of the complex, with conserved homologs found in all Gram-negative bacteria. Crystal and EM structures have caught the BamA barrel and its components within the complex in multiple conformations. Importantly, one structure shows the BamA barrel closed on its extracellular face and open to the periplasm, whereas a second has the barrel distorted, with displacement of several extracellular loops and a separation of the ß1 and ß16 strands. This lateral open state leaves the barrel open to the outer leaflet of the OM. These lateral open and closed structures are thought to represent at least two functional states in the BAM folding pathway.

The mechanism of Bam-mediated ß-barrel folding and insertion into the OM is still under intensive investigation (Hagan et al., 2011; Noinaj et al., 2015, 2017). Briefly, it is thought that after delivery of unfolded OMPs to the periplasmic complex, a rotation of the ring-like structure leads to opening of the unstable junction between the first (1st) and last (16th) ß-strands of the ß-barrel, through which the folding OMP passes en route to its final location in the OM bilayer. Supporting this conclusion, an engineered disulfide bond linking the ß-1 and ß-16 strands, thereby preventing the opening of the lateral gate, is lethal in *E. coli* (Noinaj et al., 2014).

One hypothesis is that the physical properties of the BAM complex and its interaction with the membrane bilayer may create a thinner less densely packed bilayer near to the ß1-ß16 strand-junction, thereby destabilizing the membrane to assist folding and insertion of the new ß-barrel OMP (Noinaj et al., 2013; Fleming, 2015). In this way, the BAM complex would act like a membrane disruptase to promote ß-barrel folding and insertion. The POTRA domains, likely aided by other components of the BAM complex, appear to provide a recognition site for initial binding of their unfolded OMP targets (Kim et al., 2007).

The conserved machinery for the biogenesis of ß-barrel membrane proteins is also found in mitochondria and chloroplasts. The sorting and assembly machinery (SAM) of mitochondria is required for folding and insertion of ß-barrel precursors into the mitochondrial OM. The mitochondrial homolog of BamA is called Sam50, which appears to function with a similar folding mechanism. Recent investigations of ß-barrel folding by the mitochondrial SAM complex suggest that folding precursors adopt transient ß-hairpin-like elements (Höhr et al., 2018). It is proposed that after translocating into the lumen of the barrel they may insert as ß-hairpin structures into the lateral opening between the ß1 and ß16 strands of Sam50 (Figure 4) (Höhr et al., 2018). Further, strands of the precursor, also in ß-hairpin-like structures, are then inserted sequentially into the widening lateral gate, until the entire new folded barrel can bud out from the barrel into the OM. The BamA-induced membrane thinning mentioned above may contribute to ß-barrel folding in bacteria by facilitating insertion of the new barrel into the membrane bilayer. ß-Hairpin structures and ß-strand augmentation clearly play a key role in each step of this folding mechanism.

In other work, it was shown that the BAM complex together with other ß-barrel proteins are not uniformly distributed in the bacterial OM, but rather are located in clusters or “islands” (Rassam et al., 2015). This implies that many (or most) ß-barrel proteins exist in a unique clustered environment in the bacterial OM, close to the site of folding and insertion mediated by the BAM complex. Given its essential role in ß-barrel assembly and location in the OM, it is not surprising that the BAM complex has attracted interest as a target in the search for inhibitors with antimicrobial activity.

The first inhibitor of OMP folding by the BAM complex was reported recently (Hagan et al., 2015). The inhibitor comprised a 15-mer peptide with a sequence derived from a region close to the C-terminus of BamA. The background to this study was that BamD was shown to bind to unfolded BamA. More detailed *in-vitro* assays with the BAM complex reconstituted into proteoliposomes revealed that the sequence 765–779 in BamA, as a linear peptide, binds to BamD and inhibits folding of full-length ß-barrel OMPs by the BAM complex. Moreover, a peptide containing this conserved sequence, when expressed with a signal sequence and a FLAG tag in *E. coli*, was shown by photolabeling to interact *in-vivo* with BamD, and produces growth, OM permeability and OMP biogenesis defects. At this stage, the minimal peptide sequence identified is not expected to show antimicrobial activity on whole cells, due to its inability to traverse the OM. However, this study opens the possibility of finding peptidomimetic antibiotics that exploit this mechanism to interfere with OMP assembly.

Unfolded ß-barrel proteins en route to the BAM complex contain a signal sequence (ß-signal), typically in a region corresponding to the C-terminal ß-strand, which targets them for initial interaction with the BAM complex (Robert et al., 2006; Noinaj et al., 2017). Although not a strictly conserved sequence, the ß-signal typically contains a characteristic array of hydrophobic (h) and intervening non-conserved polar (p) residues, such as Ghphph (Robert et al., 2006). In the context of a ß-sheet or ß-hairpin backbone conformation, this motif places the hydrophobic residues on one face, and polar residues on the other of the ß-sheet/ß-hairpin. A cyclic peptide was reported recently that functions as a mimetic of the ß-signal involved in targeting unfolded ß-barrel OMPs to the mitochondrial SAM complex (Jores et al., 2016). Using *in-vitro* assays, a 25-residue cyclic peptide containing the ß-signal was shown to inhibit the import of ß-barrel proteins into mitochondria. NMR analysis of the cyclic peptide suggested that it was more structured than a corresponding linear sequence. Photoaffinity cross-linking studies with isolated mitochondria, confirmed interactions of the peptide with several components of the folding complex. This work further indicates the potential of targeting ß-barrel assembly machines using peptides and suitably designed folded peptidomimetics.

Another cyclic ß-hairpin-shaped peptide called JB-95 (Figure 2) was reported recently that shows low micromolar MICs against *E. coli*, and an ability to interact with the BAM complex (Urfer et al., 2016). This ß-hairpin-shaped cyclic peptide arose from studies of macrocyclic ß-hairpin peptidomimetics containing a hairpin-inducing D-Pro-L-Pro template. The sequence attached to the template (WRIRIrWKRLRR) resembles in its distribution of polar and hydrophobic residues the ß-signal recognized by the BAM complex (Robert et al., 2006; Noinaj et al., 2017). The cyclic peptide exhibits no significant lytic activity on red blood cell membranes, but a variety of whole cell assays revealed an ability to selectively disrupt the OM but not the IM of *E. coli*. The lack of permeabilizing activity on the IM is consistent with the lack of general lytic activity on glycerophospholipid bilayers but leaves unanswered how the OM is selectively targeted. Whole cell staining with a fluorescently labeled derivative of JB-95 revealed a punctated staining pattern, consistent with selective labeling of discrete islands or patches of OMPs across the cell surface. Photochemical labeling experiments performed *in-vivo* with *E. coli* revealed cross-linking to several ß-barrel OMPs, including BamA, LptD, BtuB, and others. The binding of JB-95 to BamA/LptD in OM clusters or “islands” might therefore explain both the punctated fluorescence staining pattern and the photochemical labeling results. Finally, a proteomic analysis revealed that the peptide causes significant depletion of many OMPs (but not BamA) from treated cells. The lack of an effect on the levels of BamA in treated cells might be explained by up-regulation of *bamA* expression by an envelope stress response (σ^E^). While the results reported are consistent with inhibition of the BAM complex by JB-95, direct proof for this is still outstanding.

The exposed surface loops of BamA are potentially of interest in vaccine design as well as for antibody-based anti-Gram-negative therapeutic agents. However, bacteria use the complex carbohydrate structures in LPS as well as capsular polysaccharides to shield potential protein epitopes that might be targeted by the immune system. Nevertheless, a monoclonal antibody (mAb) called MAB1 was described recently, directed against BamA in *E. coli* (Storek et al., 2018). MAB1 was isolated from a large library of anti-BamA IgG mAbs by screening for growth inhibitory effects on an *E. coli* mutant (Δ*waaD*) displaying a truncated LPS. MAB1 showed a bactericidal activity against this strain, in the low nanomolar range over the course of several hours. Indirect evidence that MAB1 inhibits BamA folding activity was indicated by the significantly lowered levels of several OMPs in *E. coli* in the presence of MAB1. The mAb also caused activation of the σ^E^ stress response. Evidence for a permeabilizing effect of MAB1 on the cell envelope was shown by enhanced uptake of ethidium bromide, a rapid loss of periplasmic mCherry and a slower release of cytoplasmic Green Fluorescent Protein (GFP) from cells exposed to MAB1. Epitope mapping studies revealed that MAB1 interacts with residues in BamA extracellular loop 4 (light blue in Figure 4) that are distally located from the lateral gate in the ß-barrel, the POTRA domains, the BamBCDE lipoproteins and periplasmic chaperones. Further studies suggested that the ability of MAB1 to antagonize BamA function seems to correlate with structural changes to LPS that influence membrane fluidity. A direct effect of MAB1 on BamA folding activity, which is expected to be toxic for the bacteria, might be caused by an allosteric mechanism, perhaps by affecting key conformational changes required for BamA to fulfill its function as a folding catalyst.

A natural antimicrobial peptide has also been discovered that targets BamA. The lectin-like bacteriocin called LlpA, a protein of ca. 28 kDa, mediates killing of selective Gram-negative bacteria, by a mechanism that is not yet fully defined. However, BamA was recently identified as a receptor for LlpA-mediated killing in *Pseudomonas* spp. (Ghequire et al., 2018). LlpA-resistant mutants were shown to carry mutations mainly in BamA surface loop L6 (Figure 4) or genes involved in capsular polysaccharide (LPS) biosynthesis. Genetic analyses also showed that *bamA* mediates the killing effects of LlpA on sensitive strains. The results suggest that LlpA interacts with BamA loop 6, and this is facilitated and/or stabilized by interaction of the lectin-like domains of LlpA with specific carbohydrate moieties in LPS. The crystal structure of LlpA from *Pseudomonas* sp. shows the protein to be rich in ß-structure, with two mannose-binding lectin domains and a C-terminal ß-hairpin extension (Ghequire et al., 2013). A mechanism for killing by LlpA was suggested, which involves interference of the gating dynamics of BamA by the ß-hairpin loop extension, leading to the accumulation of unfolded OMPs in the periplasm and to associated downstream responses. Interestingly, BamA was also identified earlier as a receptor for ligands mediating contact-dependent growth inhibition in *E. coli* (Aoki et al., 2008), a phenomenon whereby bacterial cell growth is regulated by direct cell-to-cell contact. Again residues in loop L6, as well as L7, were implicated in BamA targeting (Ruhe et al., 2013).

These examples demonstrate that molecules interacting with the exposed external loops of BamA may have antimicrobial activity. Conceivably, molecules smaller than mAbs might be developed with similar mechanisms of action, including not only engineered antibody fragments but also smaller folded peptidomimetics better suited to circumventing the bacterial carbohydrate shield around the OM. Although one swallow does not make a summer, the intriguing discoveries highlighted in this review provide encouragement and starting points for the development of a new generation of antimicrobial agents specifically targeting Gram-negative pathogens. The need and scope for innovation in this area certainly seems to be large.

## Author Contributions

The author confirms being the sole contributor of this work and has approved it for publication.

### Conflict of Interest Statement

The author declares that the research was conducted in the absence of any commercial or financial relationships that could be construed as a potential conflict of interest.
